# A Comparison of Skeletal Muscle Diffusion Tensor Imaging Tractography Seeding Methods

**DOI:** 10.1002/nbm.70163

**Published:** 2025-10-16

**Authors:** Bruce M. Damon, Roberto Pineda Guzman, Carly A. Lockard, Xingyu Zhou

**Affiliations:** ^1^ Carle Clinical Imaging Research Program Stephens Family Clinical Research Institute, Carle Health Urbana Illinois USA; ^2^ Department of Biomedical Engineering Vanderbilt University Nashville Tennessee USA; ^3^ Department of Radiology and Radiological Sciences Vanderbilt University Nashville Tennessee USA; ^4^ Department of Bioengineering University of Illinois at Urbana‐Champaign Urbana Illinois USA; ^5^ Department of Biomedical and Translational Sciences University of Illinois at Urbana‐Champaign Urbana Illinois USA; ^6^ Beckman Institute for Advanced Science and Technology University of Illinois at Urbana‐Champaign Urbana Illinois USA

**Keywords:** DTI, fiber‐tracking, freeware, muscle architecture, simulation, skeletal muscle

## Abstract

The internal arrangement of a muscle's fibers with respect to its mechanical line of action (muscle architecture) is a major determinant of muscle function. Muscle architecture can be quantified using diffusion tensor magnetic resonance imaging‐based tractography, which propagates streamlines from a set of seed points by integrating vectors that represent the direction of greatest water diffusion (and by inference, the local fiber orientation). Previous work in skeletal muscle has demonstrated that tractography outcomes are sensitive to the method for defining seed points, but this sensitivity has not been fully examined. To do so, we developed a realistic simulated muscle architecture and implemented three methods for tract seeding: seeding along the muscle‐aponeurosis boundary with an updated procedure for rounding seed points prior to lookup in the muscle boundary mask and diffusion tensor matrices (APO); voxel‐based seeding throughout the muscle volume at a uniform spatial frequency (VXL); and seeding near external and internal muscle boundaries (EDGE). We then implemented these methods in example human datasets. The updated aponeurosis seeding procedures allow more accurate and robust tract propagation from seed points. The voxel‐based seeding methods had quantification outcomes that closely matched the updated aponeurosis seeding method. Further, the voxel‐based methods can accelerate the overall workflow and may be beneficial in high throughput analysis of multi‐muscle datasets. Continued evaluation of these methods in a wider range of muscle architectures is warranted.

## Graphical abstract

Diffusion‐tensor imaging (DTI) fiber tracking can be used to quantify skeletal muscle architecture, allowing studies of muscle structure‐function relationships. However, the options for defining the seed points (starting points for integrating the first eigenvector of the diffusion tensor) to form the fiber tracts have not been evaluated. We used simulation and experimental approaches to evaluate the impact of three seeding strategies on the muscle architecture estimates and the uniformity with which the fiber tracts sample the muscle volume.

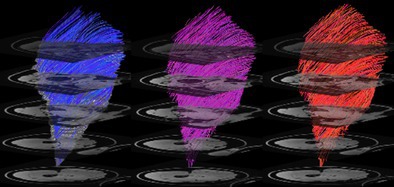

**Abbreviations**
APO: updated aponeurosis‐based seeding scheme
b‐value: diffusion‐weighting value
**D**
: diffusion tensor matrix
DT‐MRI: diffusion tensor magnetic resonance imaging
**E**
: eigenvector matrix
FA: fractional anisotropy
FOV: field of view
ICC: intraclass correlation coefficient
**L**
: eigenvalue matrix
L_FT_
: fiber tract length
MRI: magnetic resonance imaging
N_EX_
: number of excitations for signal averaging
r: diffusion‐encoding direction vector
RESOLVE: REadout Segmentation Of Long Variable Echo‐trains
S: observed signal
S_0_
: equilibrium signal intensity
SNR: signal‐to‐noise ratio
ST: slice thickness
TE: echo time
TR: repetition time
VIBE: volume interpolated breath‐hold examination
VXL: voxel‐based, regularly spaced seeding scheme
EDGE: voxel‐based, edge seeding scheme
α: angle formed by the muscle's fibers and its mechanical line of action
β: angle formed by the muscle's aponeurosis and its mechanical line of action
γ: angle formed by the muscle's fibers and its aponeurosis
ε_N_
: eigenvector of the diffusion tensor, with *N* = 1, 2, or 3
θ: angle of elevation
κ: curvature
λ_N_
: eigenvalue of the diffusion tensor, with *N* = 1, 2, or 3
ϕ: angle of azimuth

## 1 Introduction

Skeletal muscle architecture, defined here as the number and geometric properties of a muscle's fibers with respect to its mechanical line of action, impacts several aspects of skeletal muscle contraction. For example, muscle fiber length and orientation impact muscle force production, length excursion, and shortening or lengthening velocity [1, 2, 3], while muscle fiber curvature may influence the magnitudes and spatial patterns of intramuscular fluid pressure [2, 4, 5] and strain [6] development. Brightness‐mode ultrasound [7] and diffusion‐tensor MRI (DT‐MRI) tractography [8] have been used to study muscle architecture. Because MRI can also spatially map several of the functional properties of muscle contraction, there is special interest in developing DT‐MRI tractography as a quantitatively accurate method for studying muscle architecture and its relationship to function.

It is well established that water diffusion in skeletal muscle is anisotropic, with the direction of greatest diffusion aligned with the muscle fiber's long axis [9]. In the diffusion tensor model, the direction of greatest diffusion is represented by the tensor's first eigenvector. Deterministic tractography algorithms integrate these vectors from a set of seed points, resulting in streamlines that represent the local muscle architecture on the spatial scale of several fascicles per tract. How accurately these streamlines represent the fascicles' geometry depends on several data acquisition and analysis conditions. Recent works have examined the impact and optimal selections for slice thickness, in‐plane voxel size, signal‐to‐noise ratio (SNR), integration method, step‐size, termination criteria, and tract post‐processing methods, arriving at a set of proposed best practices for these stages of image acquisition and processing [10, 11, 12, 13, 14].

The present work continues this examination, with the specific focus being the definition of seed points. Two general approaches to seed point definition are described in the skeletal muscle tractography literature. First, tracts may be seeded at or near to the boundary of the muscle with its internal aponeurosis [15]. The advantage of this approach is that it facilitates calculations of mechanically relevant structural properties. As reported here, the aponeurosis‐based seeding approach allows estimation of the angles formed by (1) α, the muscle's fibers and its mechanical line of action (the pennation angle); (2) β, the aponeurosis and the muscle's mechanical line of action (as defined in Ref. [16]); and (3) γ, the muscle's fibers and its aponeurosis (often used to approximate the pennation angle). To define the aponeurosis geometry, manual definition [15] or automated selection based on signal thresholds [17] or diffusion properties [18] can be used. A disadvantage to aponeurosis‐based seeding is that partial volume artifacts between the aponeurosis of fiber insertion and the muscle may cause mis‐estimation of the diffusion tensor at the seed point. Also, digitization errors or the rounding of the decimal‐valued digitized points to integer‐valued indices for lookup into the imaging matrix may result in seed points placed in the aponeurosis rather than the muscle. To avoid this, the seeding mesh may be translated away from the true muscle‐aponeurosis boundary and into the muscle tissue. However, this procedure may result in fiber tract lengths that underestimate the true fascicle length.

As discussed in Ref. [19], other seeding approaches include placing seed points in voxels throughout the entire muscle volume or in specified imaging planes, typically corresponding to image slices. However, planar seeding methods that use too few slices may lead to incomplete muscle coverage [19, 20]. These voxel‐based seeding approaches do not require the aponeurosis to be defined; and if it is defined, the propagated tracts do not necessarily intersect with it. Therefore, this method only supports calculation of the angle α. However, a voxel‐based approach may facilitate the analysis of multi‐muscle datasets, while still providing sufficient information for many musculoskeletal modeling applications.

A search of muscle DT‐MRI tractography literature for the last 5 years revealed five publications that used aponeurosis‐based seeding, six publications that used some form of whole muscle volume voxel‐seeding, and 15 that did not specify a seeding method. However, we were unable to find any publications that compared these seeding approaches, leaving the question of how quantitative muscle architecture estimates from DT‐MRI tractography may be affected by the seeding method still unresolved. Therefore, the goals of this study were to implement and evaluate three methods for defining seed points in a muscle. The first method was aponeurosis‐based seeding as we have previously described, but with a modified procedure for looking up the diffusion tensor at the seed point to prevent complications caused by digitization errors or rounding conventions. We also evaluated two voxel‐based seeding options, regularly spaced seed points placed throughout the muscle volume and an edge‐seeding method in which seed points were placed one voxel internal to the muscle's boundaries. We first implemented and evaluated these approaches using simulated datasets. The tractography outcomes were evaluated in terms of their agreement with the ground truth fiber orientations at the voxel level, the correct termination of tracts at the muscle's outer boundary, and noise sensitivity. We subsequently demonstrated the methods' implementation in two example human datasets.

## 2 Methods

### 2.1 Simulations

#### 2.1.1 Definition of Model Tissue and Noise‐Free Images

**FIGURE 1:**
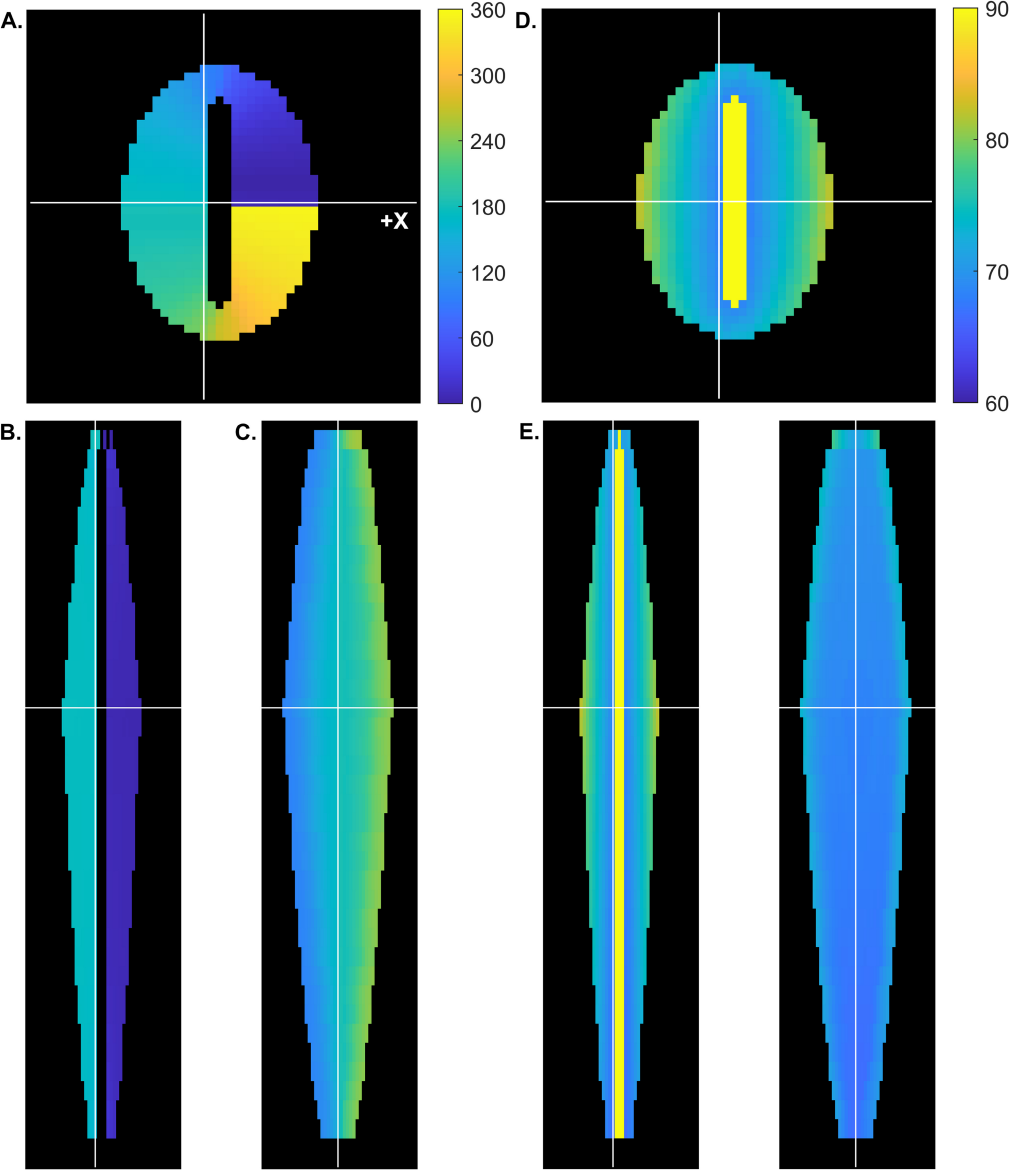
Composite tissue design and fiber orientations in the simulated muscle. (A–C) Angles of azimuth (ϕ), shown as counterclockwise rotations from the +X axis. (A) Axial view. The elliptical‐profiled tissue had a central aponeurosis in which the collagen orientation was perpendicular to the slice plane. White lines show the locations of the coronal (horizontal line) and sagittal (vertical line) views. The color bar gives ϕ in degrees. (B) Coronal view. The same color scale as in Panel (A) is used. White lines give the locations of the axial (horizontal line) and sagittal (vertical line) views. (C) Sagittal view. The angle of decreased as a function of increasing slice number. The same color scale as in Panel (A) is used. White lines give the locations of the axial (horizontal line) and coronal (vertical line) views. (D–F) Same as (A)–(C), except that the angle of elevation above the slice plane (θ) is shown. (D) Axial view. The color scale indicates the θ in degrees. θ increased as a function of within‐slice distance from the aponeurosis. (E–F) Coronal and sagittal views. The same color scale as (D) is shown. θ increased with increasing slice number.

The simulation strategy was adapted from previously described methods [10, 14, 21] and is described in detail in Section S1. The model muscle had an approximately bipennate architecture, including two approximately symmetric muscular halves placed about a central aponeurosis (Figure 1). Its design resulted in heterogeneity in the angles of muscle fiber elevation (θ) and azimuth (ϕ) and in the architectural properties of fiber length (L_FT_), pennation angle (α), and curvature (κ). Detailed procedures for modeling fiber orientations are given in Section S1. Briefly, along the long axis of the tissue (taken as the Z direction), 37 slices of tissue were defined with an in‐plane resolution of 1 × 1 mm^2^ and 6‐mm thickness. The fibers projected radially from points along the aponeurosis to corresponding points on the muscle's outer boundary (causing ϕ to vary over 360°; Figure 1A–C). The θ increased as a function of ascending slice number and as a function of distance from the aponeurosis (ranging from 66.4–82.0°, mean, 72.4°; Figure 1D–F). In each voxel, the unit‐length fiber orientation vector was taken as ε_1_, the first eigenvector of the diffusion tensor **D**. ε_3_ was calculated as the cross product of ε_1_ and a unit vector in the +Z direction and ε_2_ was calculated as the cross product of ε_1_ and ε_3_. For each muscle voxel, **D** was calculated as:

(1) $$D=E\;L\;E^{T}$$

With **E** the eigenvector matrix, **L** a diagonal matrix of eigenvalues, and the superscript *T* indicating matrix transposition. The eigenvalues of **L** were assumed to be 2.1, 1.6, and 1.4 × 10^−5^ cm^2^/s.

Noise‐free images were calculated for a matrix size of 50 × 50 and 40 slices, with one slice proximal to the muscle and two slices distal to the muscle being padded with zeros. Diffusion‐encoding used a nondiffusion weighted image and 12 diffusion‐weighted images, defined using a diffusion‐weighting (b‐) value of 475 cm^2^/s and diffusion‐encoding directions defined by the minimum energy arrangement of points on a sphere [22]. The signals were calculated as:

(2) $$S=S_{0}e^{-b\;r\;D\;r^{t}}$$

With *S* the diffusion‐weighted signal, *S*_*0*_ the nondiffusion‐weighted signal (=1), and *r* a three‐element vector specifying each diffusion‐encoding direction.

#### 2.1.2 Updated Aponeurosis Seeding Scheme: Noise‐Free Condition

An updated method for initiating fiber tracts from the points defining a seeding mesh was defined and implemented. Specifically, we introduce the following procedure to ensure that any seed point placed within one voxel of the muscle‐aponeurosis boundary can be used to initiate a fiber tract, while still beginning at the preferred seed point. The method is based on the assumption that the spatial frequency of variations in muscle fiber orientation is low enough that the diffusion tensor from any voxel containing muscle tissue neighboring the voxel of interest can be used to estimate the diffusion tensor within the voxel of interest:

1. Form masks defining the internal aponeurosis boundaries and the muscle boundaries (the latter excluding the internal aponeurosis).
2. Define an aponeurosis seeding mesh at the muscle‐aponeurosis boundary. Define each vertex in this mesh as a seed point and find its row, column, and slice coordinates.
3. Round the slice coordinates using standard rounding conventions; for the row and column coordinates, take both the floor and ceiling.
4. For each of the four combinations of integer row and column coordinates, determine if the point is included within the muscle boundary mask or the aponeurosis boundary mask.
5. For each point included within the muscle boundary mask, determine the distance between the rounded coordinates and the seed point; define the point with the smallest distance as the closest included point.
6. Propagate a tract from the seed point using the diffusion direction found upon lookup using the closest included point.

These procedures are illustrated in Section S2.

Fiber tracking was performed using the MuscleDTI_Toolbox [23], as previously described [14, 17, 24] except as updated for this work; see Section S3, Table S1 for a list of function versions used and their modifications from previous publications. Fiber tracts were propagated from the seed points in the +Z direction by Euler integration of ε_1_ at a step‐size of one voxel‐width. Premature termination due to excessive inter‐point angles or fractional anisotropy (FA) values did not occur in the noise‐free condition. To smooth the tracts, their row, column, and slice coordinates were fitted to 2nd‐, 2nd‐, and 3rd‐order polynomials, respectively, as functions of point number.

The smoothed tracts' architectural properties were characterized as follows. Tracts with length (L_FT_) < 5 mm were excluded. To promote uniform sampling throughout the muscle volume, the farthest streamline sampling (FSS) method described by Li et al. [25] was implemented, with sample sizes of 1000 and 500 tracts preserved from the original dataset (FSS1000 and FSS500, respectively). The central axis along the longest direction of the muscle, taken as the mechanical line of action, was determined by fitting 3rd‐order polynomial functions through the row and column coordinates of each muscle slice's centroid, as a function of slice number. For each preserved tract, the α values were calculated in a pointwise manner along the tract by finding the angle formed by the line connecting the fiber‐tract point to the seed point and the local tangent to the central axis at the level of the seed point. The tangent was calculated as a unit‐length vector describing the slice‐wise difference in the central axis position. Curvature (κ) was calculated in a pointwise manner along the tract using the Frenet‐Serret formulas, as previously described [21]. The tracts were characterized with their average α, average κ, and L_FT_ (the last calculated as the summed Euclidean distance between points). For each architecture property, the whole‐muscle mean value was calculated.

The fiber orientations within the tracts were evaluated by comparing the α for each segment of the smoothed fiber tracts to the ground truth data at the corresponding voxel location. The mean angular difference and limits of agreement for the whole muscle were calculated and visualized using Bland–Altman plots [26], and the type 3,1 intraclass correlation coefficients (ICCs) were calculated between the estimated and ground truth angles. To characterize the uniformity of the sampling of the muscle volume by the fiber‐tracts, we first calculated the number of fiber tract points in each voxel within the muscle boundary mask. Then, the whole‐muscle mean, standard deviation (SD), and coefficient of variation (CV) of the points/voxel were calculated, and the percentage of voxels containing at least one fiber tracking point over the entire muscle volume (% Filled) was calculated. Also, the mean and SD of points/voxel for each slice were calculated.

#### 2.1.3 VXL and EDGE Seeding Schemes: Noise‐Free Condition

Two methods of voxel‐based seeding were evaluated: regularly spaced seeding throughout the muscle volume (VXL) and edge (EDGE) seeding. For VXL, seed points were placed in every second voxel in the row and column directions, within a once‐eroded muscle boundary mask. For EDGE, seed points were placed in all voxels that lay along the outer edge of the eroded muscle mask and along the outer edge of the dilated aponeurosis boundary mask. These seeding methods are illustrated in Section S4.

Fiber‐tracts were propagated bidirectionally from the seed points. Premature termination did not occur in the noise‐free condition; however, rounding and tract termination conventions sometimes resulted in tract propagation into the internal aponeurosis region of the muscle. Therefore, up to four innermost points of each fiber tract were removed if they lay inside of the internal aponeurosis boundaries. Tract smoothing was performed as described as in Section 2.1.2. Tracts with L_FT_ < 5 mm were excluded. FSS1000 and FSS500 were implemented as described in Section 2.1.2. Fiber‐tract architecture characterization included the L_FT_, mean α, and mean κ, with α calculated by treating the innermost point on the fiber tract as the seed point. The same whole‐muscle statistics as described for APO seeding were calculated.

#### 2.1.4 Comparison of Seeding Schemes Using Noisy Data

The performance of the APO, VXL, and EDGE seeding methods was evaluated in the presence of noisy data. The following procedures were performed at SNR levels of 39 and 54, with these SNR levels being typical for in vivo studies and corresponding to previous in silico models used to test muscle DT‐MRI tractography methods [14]. For each of 1000 independent trials, Gaussian‐distributed noise was added to the noise‐free images, as previously described [14]. The noisy images were used to estimate **D** using a weighted least squares method, followed by singular value decomposition of **D** to obtain **E** and **L**. Fiber tracts were propagated and analyzed using the procedures described above, except that premature tract termination could occur if either the FA value fell outside range of 0.1–0.4 or the angle between the propagated segment and the second segment preceding the propagated segment exceeded 30° for two consecutive points. Fiber‐tracts with L_FT_ < 5 mm were excluded. The remaining fiber‐tracts were smoothed as described as in Section 2.1.2, sampled using FSS1000 and FSS500, and characterized with their L_FT_, average α, and average κ. These data were averaged across the entire muscle. At the end of each of the 1000 trials, the average value of each parameter was calculated. After the 1000th trial, the population mean was calculated and the 95% confidence intervals were formed. The outcomes were evaluated by comparing the architectural properties to those of the noise‐free APO condition.

### 2.2 Demonstration in Human Data

The performance of the seeding methods was demonstrated with in vivo data. These procedures were approved by our local Institutional Review Board, for which a 30‐year‐old male volunteer provided voluntary informed consent. The tibialis anterior (TA) was selected as an example bipennate muscle and the gluteus maximus (G_Max_) was selected as an example multipennate muscle. MRI used a Siemens Vida, XA60 3T MRI scanner with 45 mT/m maximum amplitude, 200 T/m/s maximum slew rate gradients. The participant was positioned supine on a patient bed containing built‐in spine elements. The torso, thigh, and heel were supported to raise the pelvis and leg above the patient bed. For the TA study, an 18‐element array coil was placed over the anterior surface of the leg and supported by a frame running along the leg's medial and lateral surfaces. For the G_Max_ study, an 18‐element array coil, supported by the frame, was placed over the anterior pelvis. The body coil was used for transmission; receive coil selection used the manufacturer's “Auto Coil Select” function.

For both muscles, structural imaging data were acquired using Siemens' product implementation of quantitative fat‐water imaging (Volume Interpolated Breath‐hold Examination, VIBE) based on a three‐dimensional (3D) Fast Low‐Angle SHot gradient echo acquisition. DT‐MRI data were acquired using a REadout Segmentation Of Long Variable Echo‐trains (RESOLVE) sequence. The parameters for both sequences are given in Table 1. For each type of image, slices were acquired in three stacks, with four overlapping slices for VIBE and two overlapping slices for RESOLVE. For each image stack, the shim volume was defined to cover the muscles in each stack, while minimizing fat and air coverage. The full width at half maximum peak height was measured prior to each RESOLVE scan, with values of 46.6, 49.5, and 62.7 Hz for the stacks in the TA and 45.3, 60.2, and 31.4 Hz for the stacks in the G_Max_.

**TABLE 1:** Imaging sequence parameters.

Muscle	Sequence	TR (ms)	TE (ms)	Flip angle (°)	Diffusion encoding	Slices	Matrix	FOV (mm × mm)	N_EX_	Readout segments
TA	VIBE	9.23	[1.37, 2.60, …, 7.52]	4		3, 40, 3	160 × 160 (320 × 320)	200 × 200	1	
	RESOLVE	5000	45.18		450, 11.0, 21.2	3, 20, 6	72 × 72 (144 × 144)	200 × 200	4 (b = 0) 2 (b = 450)	5
GM	VIBE	9.23	[1.37, 2.60, …, 7.52]	4		4, 40, 3	160 × 160 (320 × 320)	256 × 256	1	
	RESOLVE	5000	45.18		450, 10.8, 21.9	3, 40, 3	64 × 64 (128 × 128)	256 × 256	4 (b = 0) 2 (b = 450)	5

*Note:* Matrix sizes are given as acquired (reconstructed). Under the Diffusion Encoding heading, the data presented include the b‐value (in s/mm^2^) and δ and Δ in ms. Under the Slices heading, the data presented include the number of stacks, number of slices per stack, and slice thickness in mm. All acquisitions used contiguous slices. For DT‐MRI, the diffusion gradient duration and separation were 21.9 and 10.8 m, respectively.

Abbreviations: b‐value, diffusion‐encoding value for diffusion‐weighted images in s/mm^2^; FOV, in‐plane field of view; N_EX_, number of excitations for signal averaging; TE, echo time; TR, repetition time; δ, diffusion gradient duration in ms; Δ, diffusion gradient separation in ms.

The VIBE data were processed using the manufacturer's algorithms to produce maps of the fat and water signal distributions. For both VIBE and RESOLVE, an offset was observed between the corresponding slices in the overlapping regions of the proximal and distal image stacks. The offset was corrected by registering the first and second stacks of the VIBE and RESOLVE datasets with a 2D rigid body translation using MATLAB's *imwarp()* function. The interstack 2D translations were defined using the *imregtform()* function, with one transformation obtained from each pair of overlapping slices, and a final transformation defined for each dataset by averaging the 2D transformations of the overlapping slices of each respective VIBE and RESOLVE stack pair. After repeating this procedure for the second and third stacks, the redundant overlapping slices were eliminated and the stacks were concatenated. Finally, the VIBE dataset was resized to match the RESOLVE dataset, and the RESOLVE dataset was registered to the resized VIBE dataset with a 2D rigid body translation transformation. The 2D translation applied to the RESOLVE dataset was defined by averaging the 2D transformations obtained after individually registering the central 50 slices of both datasets using the *imregtform()* function. The registered RESOLVE datasets were denoised using anisotropic smoothing [27, 28] at a level of 5% [29, 30]. Boundary masks were drawn around the muscles of interest. The diffusion tensor fields in these muscles were computed from the denoised dataset using a weighted linear least squares method.

Fiber tracts were generated using the APO, VXL, and EDGE methods. For APO, the aponeurosis was segmented and formed into a seeding mesh using the *define_roi()* function in the MuscleDTI_Toolbox [17]. To capture spatial variations in muscle architecture, mesh sizes of 45 × 35 and 155 × 63 (rows × columns) were used in the TA and G_Max_, respectively, resulting in minimum seed point densities of ~1/voxel. The VXL‐ and EDGE‐based seeding schemes were implemented as described for simulated muscles, except that for VXL the seed points were placed in every third voxel in the row and column directions. Fiber tracts were propagated using Euler integration at a step‐size of one voxel‐width. Tract termination occurred at the muscle boundary or if either the FA value fell outside range of 0.05–0.40 or the angle between the propagated segment and the second segment preceding the propagated segment exceeded 30° for two consecutive points. The *fiber_smoother_v14()* function was used to smooth the remaining tracts in the row, column, and slice directions using 3rd order polynomial functions. The *fiber_quantifier()* function was used to characterize the smoothed tracts' L_FT_, average α, and average κ. In the TA muscle, fiber‐tracts with L_FT_ < 10 mm, α > 25°, and κ > 20 m^−1^ were excluded from further analysis. In the G_Max_, fiber‐tracts with L_FT_ < 12.5 mm were excluded. FSS1000 and FSS500 were applied. For APO, γ was calculated as previously described in the MuscleDTI_Toolbox [17], α was calculated as described above, and β was calculated as (α − γ). The whole muscle average values were calculated.

## 3 Results

### 3.1 Simulations

**FIGURE 2:**
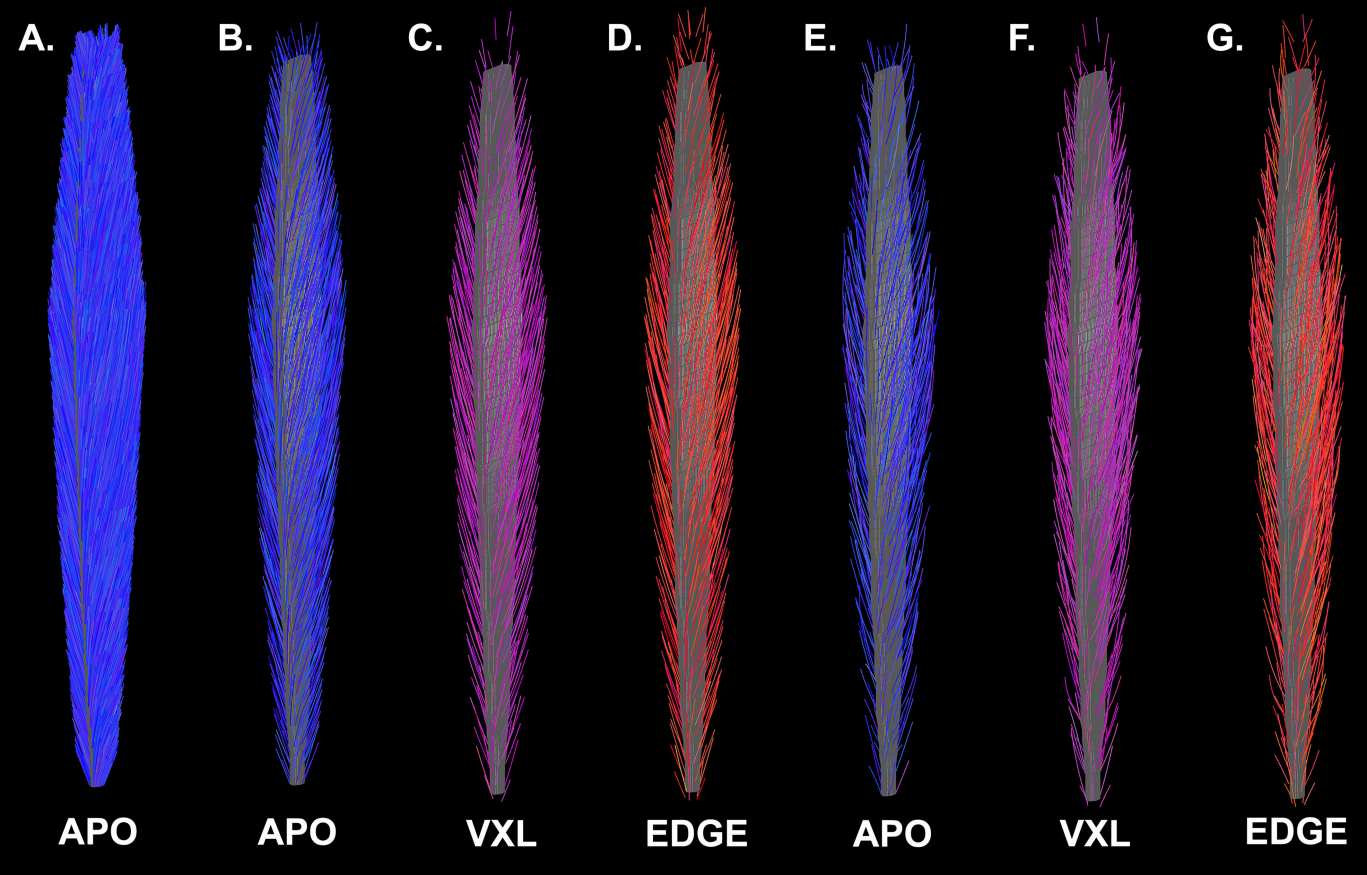
Fiber‐tracts generated under noise‐free conditions (simulated muscle). (A–D) Noise‐free simulations, with (A) All fiber tracts, APO seeding; (B) FSS500, APO seeding; (C) FSS500, VXL seeding; and (D) FSS500, EDGE seeding. (E–G) SNR = 39 simulations, with FSS500 sampling for *E. APO* seeding; (F) VXL seeding; and (G) EDGE seeding. Seeding methods are indicated by color and with labels for each panel.

Figure 2 shows example fiber‐tracts generated from the simulated muscles. Panel A shows all of the tracts propagated from APO‐seeded points. Panels B–D show the FSS500‐sampled tracts for the APO, VXL, and EDGE seeding conditions, respectively. Panels E–F show example FSS500 fiber tracts, obtained using APO, VXL, and EDGE seeding, for the SNR = 39 noise condition.

**FIGURE 3:**
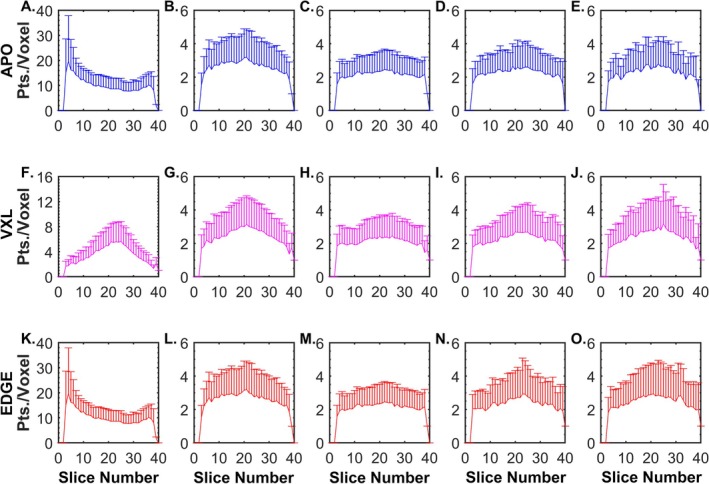
Uniformity of spatial sampling of the muscle by the fiber tracts: Effects of seeding method, noise, and sampling condition (simulated muscle). Uniformity is expressed as the variability in the average number of fiber tract points per voxel (Pts./Voxel) in each slice. (A) ***E.** APO* seeding, with (A) showing data from noise‐free images (all fibers); (B) showing data from noise‐free images (FSS1000); (C) showing data from noise‐free images (FSS500); (D) showing data from SNR = 54 and FSS500; and (E) showing data from SNR = 39 and FSS500. (F–J) Same as (A)–(E), but for VXL seeding. (K–O) Same as (A)–(E), but for EDGE seeding. All plots with SNR = 39 or 54 data show example results from the 1000th noise realization trial. Seeding methods are indicated by color and with Y‐axis labels. Note differences in Y‐axis scales.

Figure 3 shows the slice‐wise mean and SD of the number of fiber‐tracking points per voxel for the simulated muscle, with panels A–E showing data for APO seeding, F–J showing data for VXL seeding, and K–O showing data for EDGE seeding. The mean, SD, and CV over all voxels within the muscle mask, and the % Filled voxels over the whole muscle, are shown in Table 2. For all conditions, the CV decreased when FSS was used and as a function of noise level. At realistic noise levels, the CV was lower for APO seeding than for VXL or EDGE. Collectively, these data indicate that APO seeding provided the most uniform sampling of the muscle for realistic noise levels and that FSS1000 and FSS500 were associated with a more uniform sampling of the muscle volume than including all tracts.

**TABLE 2:** Sampling of muscle volume by fiber tracts: Effects of seeding method and sampling condition (simulated muscles).

Seeding method	Sampling condition	Noise level	Points/voxel	CV (%)	% Filled
APO	All	Noise‐free	9.4 (4.1)	43.2	94.0
	FSS1000	Noise‐free	2.4 (0.8)	33.7	55.7
	FSS500	Noise‐free	2.0 (0.6)	31.5	33.7
	FSS500	54	2.4 (1.3)	55.2	38.2
	FSS500	39	2.4 (1.4)	58.1	36.6
VXL	All	Noise‐free	3.3 (1.5)	47.0	79.7
	FSS1000	Noise‐free	2.3 (0.7)	31.4	63.6
	FSS500	Noise‐free	2.0 (0.5)	27.2	36.6
	FSS500	54	2.4 (1.5)	60.8	42.0
	FSS500	39	2.6 (1.7)	64.7	40.4
EDGE	All	Noise‐free	5.4 (2.1)	39.3	102.0
	FSS1000	Noise‐free	2.3 (0.6)	28.3	60.4
	FSS500	Noise‐free	2.0 (0.5)	26.9	35.2
	FSS500	54	2.5 (1.6)	64.2	40.1
	FSS500	39	2.6 (1.8)	67.5	38.2

*Note:* For points/voxel, mean (SD) is given. For SNR = 54 and 39 conditions, statistics are reported for the final trial.

**FIGURE 4:**
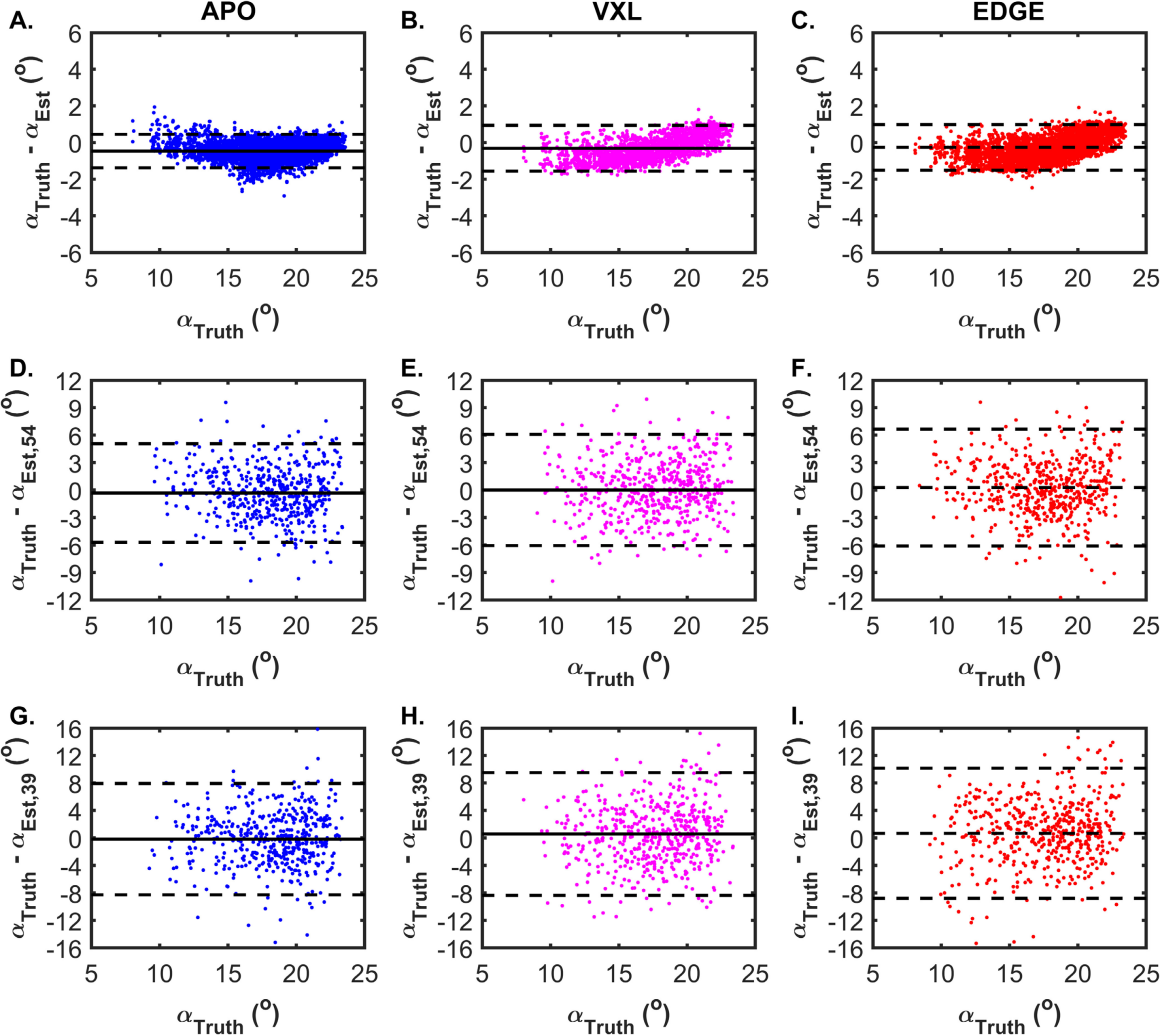
Bland–Altman plots illustrating the agreement between α angles derived directly from ground‐truth values for ε_1_ and the fiber‐tracts. (A–C) Noise‐free data, with ground truth angles and tract‐derived values. Panels (A)–(C) show APO, VXL, and EDGE seeding, respectively. (D–F) Same as (A)–(C), except for FSS500 and SNR = 54. (G–I) Same as (A)–(C), except for FSS500 and SNR = 39. Solid horizontal lines indicate the mean difference; dashed horizontal lines indicate the limits of agreement. In all plots, every 10th point is illustrated to improve clarity of presentation. All plots with SNR = 39 or 54 data show results from the 1000th noise realization trial. Seeding methods are indicated by color and with labels at the top of each column. Note differences in Y‐axis scales.

Figure 4A–C shows Bland–Altman plots indicating the agreement of the known and estimated α angles for the APO, VXL, and EDGE conditions, respectively. For VXL and EDGE, the differences were slightly upsloping over this range of values and under these experimental conditions; but overall, the level of agreement was strong, with ICC values > 0.97 and narrow limits of agreement that included zero. Panels D–F and G–I, respectively, show Bland–Altman plots for the SNR = 54 and 39 conditions, respectively, with wider limits of agreement and lower ICC values (~0.65 and ~0.5 for SNR = 54 and 39, respectively) than for the noise‐free condition, but still centered on zero.

Table 3 shows the effect of the seeding method, FSS sampling procedure, and noise level on the estimated architectural properties. For L_FT_ and under the APO seeding scheme, the mean value of all initially propagated tracts was lower than the corresponding mean values after FSS sampling; however, the opposite trend occurred for L_FT_ with the VXL and EDGE seeding schemes. No systematic effects of the sampling procedure on the mean values of α and κ were noted for any seeding strategy. For the whole‐muscle average α and κ estimates, the values were lower for VXL and EDGE seeding than for APO, while the opposite trend occurred for L_FT_.

**TABLE 3:** Whole‐muscle architecture characteristics: Effects of seeding method, FSS sampling, and noise level (simulated muscles).

Seeding method	Sampling condition	Noise level	L_FT_ (mm)	α (°)	κ (m^−1^)
APO	All	Noise‐free	18.9	20.3	6.7
	FSS1000	Noise‐free	21.4	20.1	6.6
	FSS500	Noise‐free	21.0	20.1	6.7
	FSS500	54	22.1 [21.8 22.5]	19.9 [19.7 20.1]	9.1 [8.7 9.5]
	FSS500	39	21.4 [20.9 21.9]	19.9 [19.7 20.3]	12.3 [11.6 13.2]
VXL	All	Noise‐free	25.6	19.2	5.6
	FSS1000	Noise‐free	23.3	19.5	5.5
	FSS500	Noise‐free	22.4	19.5	5.5
	FSS500	54	24.5 [24.0 25.0]	19.1 [18.9 19.4]	8.7 [8.2 9.1]
	FSS500	39	24.7 [24.0 25.3]	18.6 [18.2 19.0]	11.7 [10.9 12.6]
EDGE	All	Noise‐free	22.2	19.5	5.6
	FSS1000	Noise‐free	21.3	19.6	5.5
	FSS500	Noise‐free	21.6	19.6	5.5
	FSS500	54	23.9 [23.4 24.4]	19.2 [19.0 19.5]	9.2 [8.8 9.8]
	FSS500	39	23.9 [23.2 24.6]	18.5 [18.1 18.9]	12.9 [12.0 13.9]

*Note:* For noise‐free data, a whole‐muscle mean value is given. For SNR = 54 or 39, the mean and 95% confidence interval across 1000 independent noise realization trials are given.

### 3.2 Demonstration in Human Data

#### 3.2.1 Tibialis Anterior Muscle

**FIGURE 5:**
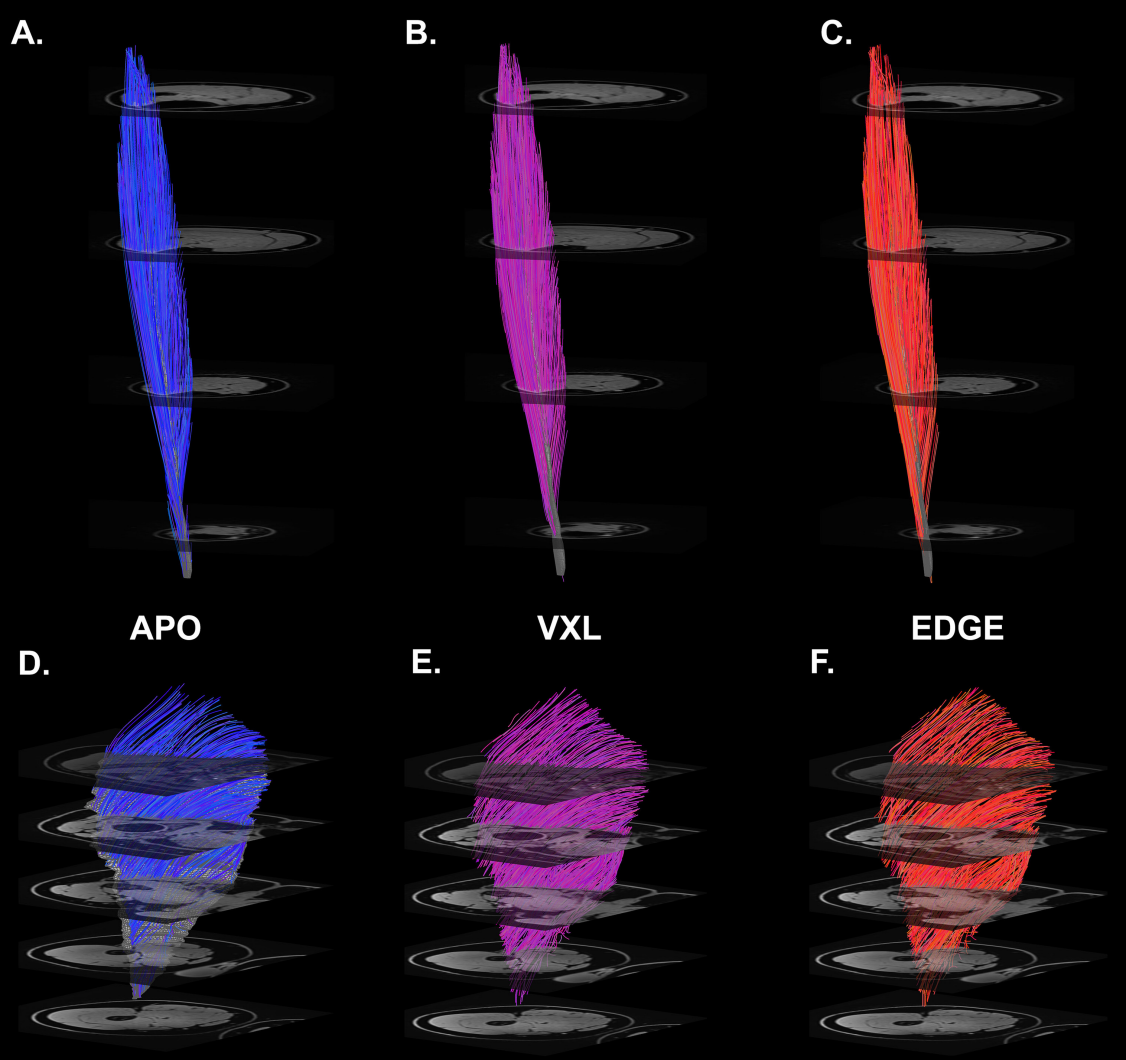
Fiber tracts from the in vivo studies. Panels (A)–(C) show fiber tracts generated using APO, VXL, and EDGE seeding, respectively, from the tibialis anterior muscle under the FSS500 sampling condition. Panels (D)–(F) show fiber tracts generated using APO, VXL, and EDGE seeding, respectively, from the gluteus maximus muscle under the FSS1000 sampling condition. Within each panel, the aspect ratio matches actual anatomical dimensions. Panels (A)–(C) have consistent scaling and panels (D)–(F) have consistent scaling. Seeding methods are indicated by color and with labels between the upper and lower rows.

**FIGURE 6:**
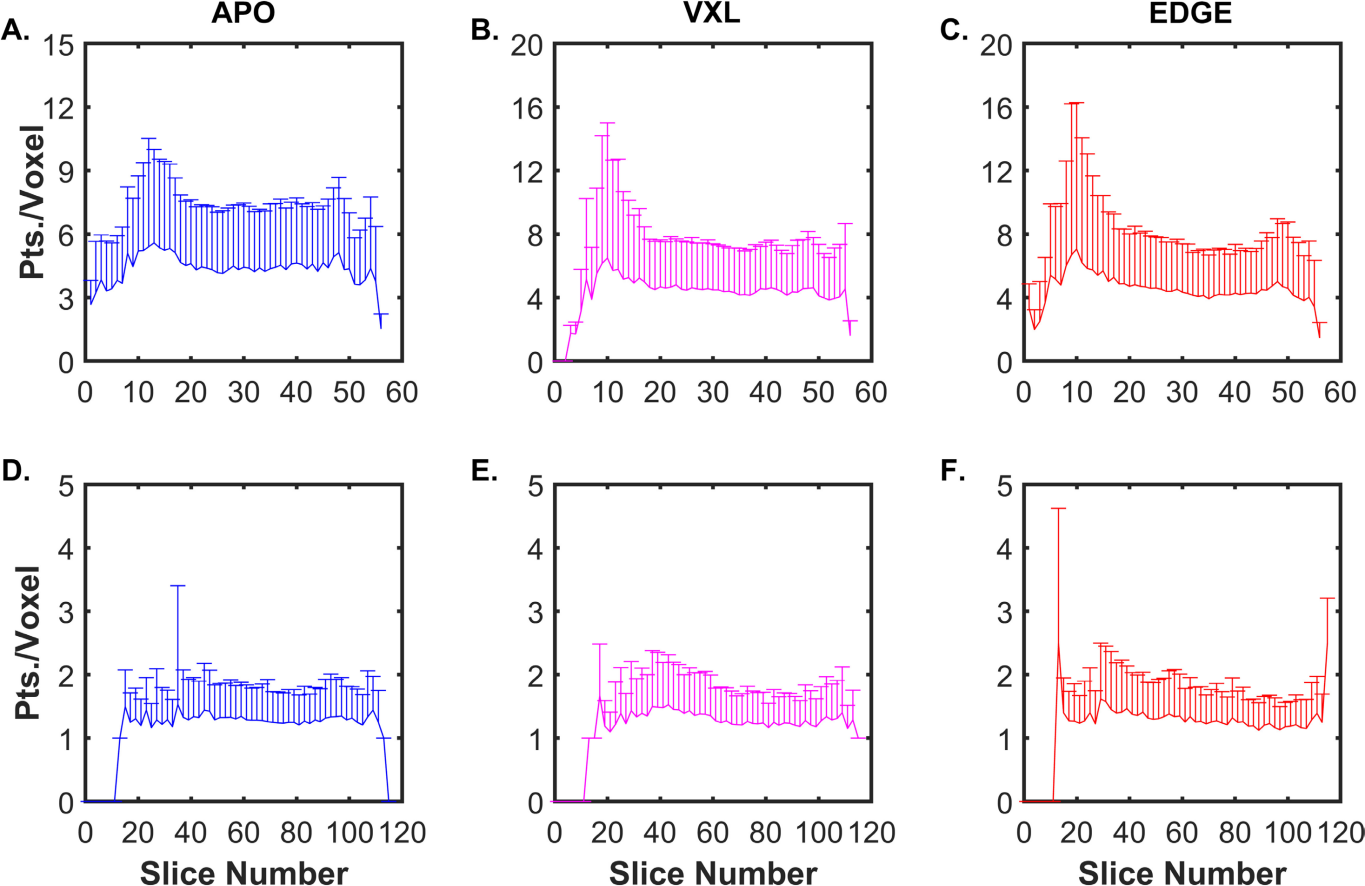
Uniformity of spatial sampling of the muscle by the fiber tracts (in vivo data). Uniformity is expressed as the variability in the average number of fiber tract points per voxel in each slice. Panels (A)–(C) show data for the TA and panels (D)–(E) show data for the G_Max_, with (A) and (D) showing data for APO, (B) and (E) showing data for VXL, and (C) and (F) showing data for EDGE. For the G_Max_, data from every other slice are plotted to improve clarity. Seeding methods are indicated by color and with labels at the top of each column. Note differences in Y‐axis scales.

The SNR of a representative b = 0 image from the TA dataset was 41.5. Figure 5A–C shows FSS500 fiber‐tracts from the TA muscle under each seeding condition. Figure 6A–C shows the slice‐wise average number and SD of fiber‐tract points per voxel for each seeding method. Table 4 reports the sampling statistics, revealing that APO had the most uniform sampling and EDGE had the least uniform sampling. In all cases, FSS sampling improved the sampling uniformity. The architecture estimates are provided in Table 5. In general, the average L_FT_ values for the VXL and EDGE conditions were slightly larger than for the APO condition, and the α and κ values were slightly higher for APO than for the VXL and EDGE schemes. The β and γ angles are reported for the APO scheme.

**TABLE 4:** Sampling of muscle volume by fiber tracts: Effect of seeding method and sampling condition (in vivo data).

Muscle	Seeding method	Sampling condition	Points/voxel	CV (%)	% Filled
TA	APO	All	8.8 (2.0)	22.6	79.2
		FSS1000	6.4 (1.1)	17.5	72.7
		FSS500	4.3 (0.9)	17.5	53.7
	VXL	All	7.4 (1.7)	23.4	83.8
		FSS1000	6.0 (1.3)	20.8	80.8
		FSS500	4.2 (0.9)	20.8	58.3
	EDGE	All	24.1 (8.7)	36.2	99.4
		FSS1000	6.5 (1.9)	28.8	76.7
		FSS500	4.6 (1.0)	28.8	53.1
G_Max_	APO	All	4.4 (2.0)	45.3	62.7
		FSS1000	1.3 (0.5)	38.6	36.9
		FSS500	1.1 (0.4)	39.5	21.0
	VXL	All	3.9 (1.9)	48.6	90.6
		FSS1000	1.3 (0.5)	36.2	44.1
		FSS500	1.2 (0.4)	35.4	24.6
	EDGE	All	8.3 (4.0)	48.1	99.3
		FSS1000	1.3 (0.5)	35.7	39.3
		FSS500	1.2 (0.5)	41.4	21.4

*Note:* For points/voxel, mean (SD) is given.

**TABLE 5:** Whole‐muscle architecture characteristics: Effects of seeding method and sampling strategy (in vivo muscles).

Muscle	Seeding method	Sampling condition	L_FT_ (mm)	α (°)	β (°)	γ (°)	κ (m^−1^)
TA	APO	All	108.2	8.1	1.3	6.8	4.3
		FSS1000	111.7	8.0	1.2	6.8	4.1
		FSS500	114.5	8.1	1.4	6.7	4.1
	VXL	All	123.4	7.2			3.3
		FSS1000	120.4	7.7			3.5
		FSS500	120.4	7.8			3.7
	EDGE	All	124.9	7.8			3.5
		FSS1000	117.2	8.0			3.8
		FSS500	116.7	8.0			3.8
G_Max_	APO	All	82.5	39.3	15.2	24.1	14.4
		FSS1000	78.0	39.0	14.2	24.7	21.6
		FSS500	72.4	39.4	16.4	23.0	29.2
	VXL	All	112.4	34.7			10.6
		FSS1000	91.2	35.5			16.1
		FSS500	85.1	36.3			17.8
	EDGE	All	93.9	37.0			13.3
		FSS1000	79.8	36.5			19.1
		FSS500	74.1	36.8			19.5

*Note:* For all data, the whole‐muscle mean is given.

#### 3.2.2 Gluteus Maximus Muscle

The SNR of a representative b = 0 image from the G_Max_ dataset was 45.4. Figure 5, panels D‐F show FSS1000 fiber‐tracts from the G_Max_ muscle. Figure 6, panels D‐F and Table 4 show sampling statistics for the G_Max_ muscle, with a similar trend being noted for the effect of FSS as in the TA muscle. However, no effect of seeding scheme on the sampling uniformity was evident. For the architecture estimates, the only trend that was evident was an increase in the mean curvature estimate when FSS was used. The β and γ angles are reported for the APO scheme.

## 4 Discussion

### 4.1 Modeling of Muscle Architecture for DT‐MRI Tractography Simulations

The simulation approach used here is based on previous uses of simulated muscle architectures for code validation and understanding the effects of image acquisition and tractography settings on the accuracy and precision of DT‐MRI tractography‐derived architecture estimates [10, 11, 14, 17, 21]. We expanded these approaches by using two mechanisms to create architectural heterogeneity. The first mechanism was the muscle's overall morphology: the muscle's minor and major radii varied as a function of slice number, creating heterogeneity in muscle fiber length. In addition, fiber orientation varied: the angles of azimuth were distributed in an approximately radial pattern and the angles of elevation increased with both ascending slice number and in‐plane distance from the center of the muscle. Importantly, properties such as fiber length and orientation heterogeneity are present in many human muscles. For example, in the vastus medialis muscle, the pennation angle increases from 5° to 50° (Ref. [31]) and the muscle thickness increases as it proceeds from origin to insertion. Moreover, Blemker et al. have shown that architectural heterogeneity can induce heterogeneous patterns of strain development during contraction [6], and recent finite element models of muscle that incorporate heterogeneous fiber architecture have been used to predict fascicle and tissue behavior in passive and active muscle lengthening [32]. The existence and functional impact of architectural heterogeneity therefore illustrates the importance of using heterogeneous architectures in simulation applications such as this, as well as developing seeding and tract post‐processing methods that promote uniform spatial sampling by the fiber‐tracts.

### 4.2 Aponeurosis Seeding Schemes

Aponeurosis seeding is an example of tract propagation from functionally relevant seed points, much like using functional MRI‐derived regions of interest as seed points for white matter tractography. For skeletal muscle tractography, the functional relevance of aponeurosis seeding is that the aponeurosis is the structure into which most of the muscle fascicles insert and through which most of the fibers' force is transmitted. As introduced here, aponeurosis‐seeding also allows the estimation of additional functionally relevant structural properties, including the aponeurosis's orientation with respect to the muscle fibers and mechanical line of action. We also introduced an updated procedure for finding the initial direction of fiber tract propagation, based on a search of the in‐plane adjacent voxels' diffusion tensors. This procedure was needed because aponeurosis‐based seeding can suffer from partial volume artifacts between muscle and aponeurosis, which could otherwise induce ε_1_ estimation errors. These errors are similar to the partial volume artifacts between white and gray matter that can occur in white matter tractography based on functionally derived seeding regions [33].

The Bland–Altman plots showed that when fiber orientation estimates were derived from the APO‐seeded tracts, there was acceptable accuracy with respect to ground truth: under all noise levels examined, the limits of agreement included zero. While there may have been a slight value‐dependent bias under the experimental conditions used, the bias was < 1° and would therefore not be expected to impact muscle architecture‐based predictions of force production significantly (which vary with the cosine of the pennation angle). As revealed by the larger limits of agreement and lower ICC values, the precision of the estimates decreased with SNR; however, the errors appeared to be random with no systematic bias evident. This finding suggests that any resulting errors in architecture estimation would be primarily manifest as overestimates of curvature and fiber‐tract length, which is consistent with our observations in Table 3.

Another source of potential error in aponeurosis seeding is that when FSS sampling was not used, the fiber‐tract point density per voxel varied in a manner that apparently corresponds to the muscle and aponeurosis cross‐sectional area in the slice. This was observed in both the simulated and human muscles. The impact of this error is likely to depend on the existence and nature of any architecture heterogeneity within the muscle; for example, if the tract density is highest in a region of the muscle having shorter fiber tracts, then these shorter tract lengths will be more heavily weighted in the calculation of a simple whole‐muscle average. However, improving the sampling uniformity using FSS substantially reduced this error. Other errors were due to noise: at SNR levels of 39 and 54, the estimated fiber tract length and curvature levels were overestimated in an SNR‐dependent manner, consistent with previous findings [14, 21].

### 4.3 Voxel‐Based Seeding Schemes

Another disadvantage to aponeurosis‐seeding is the time‐consuming nature of region definition. This is especially true for manual definition approaches, but even automated selection methods may require manual inspection and correction. The voxel‐based seeding schemes implemented here obviate the need for these manual steps. Although voxel‐seeding schemes do not allow the aponeurosis orientation with respect to the fibers or the mechanical line of action to be estimated, the information about muscle architecture that they do provide may continue to be useful in many musculoskeletal modeling applications.

The voxel‐based schemes used here are similar to whole volume seeding methods that are used in white matter tractography. Like those approaches, VXL ensures whole‐organ coverage, which might otherwise not occur if excessive FA or high inter‐point angle criteria terminate a propagating tract before it reaches a muscle boundary. But also as in white matter tractography—in which large white matter bundles may be overrepresented by a biased distribution of seed points [34]—voxel‐based seeding without using FSS resulted in nonuniform tract densities across the slices. An alternative voxel‐based seeding method to VXL and EDGE is the definition of planar sections as seeding regions. As noted by Budzik et al. [20] and as illustrated in the review by Damon et al. [19], however, it is necessary to define multiple planes when seeding based on planar regions of interest, because not all of the fibers in a pennate muscle will pass through a single anatomical plane. VXL and EDGE distribute seed points across all slices, thereby thus intrinsically avoiding this issue.

In simulated muscle, in both noise‐free and noisy images, the VXL and EDGE‐seeding methods resulted in estimates of L_FT_ (higher), α (lower), and κ (lower) that differed from those of the APO condition. Consistent with the behaviors observed in the Bland–Altman plots (including the absence of a significant bias and wider limits of agreement at realistic SNR levels) and the lower ICC values, the tract length and curvature estimates demonstrated clear SNR dependence. For human muscles, however, any differences in the architectural estimates of the VXL and EDGE conditions from the APO condition were less systematic and may have been muscle‐specific. This question should be investigated further.

### 4.4 Potential Applications of Updated Aponeurosis and Voxel‐Based Seeding Schemes

The APO, VXL, and EDGE seeding schemes may offer unique sets of advantages, disadvantages, and opportunities. For example, the updated APO seeding scheme, while time‐consuming, also enables additional applications. One possibility is its use in generating muscle mechanical models that are based on muscle‐tendon geometry. Another potential application for APO seeding relates to the recent introduction of Laplacian flow simulation to recreate muscle architecture [35] and support finite element modeling of muscle mechanics [32]. These flow simulations can be validated with DTI tractography data; however, they are sensitive to the aponeurosis definition [36]. Improved aponeurosis seeding methods may provide more robust methods to validate Laplacian flow simulations or other methods for recreating muscle architecture and modeling muscle function.

One application of voxel‐seeding may be in the analysis of large sample size, multi‐muscle datasets in which the time required for manual steps would significantly impair high throughput analysis. Another potential use of voxel‐based seeding schemes is that they may be more robust to early termination or discontinuities of fiber tracts caused by compositional heterogeneity in muscle disease, such as fibrosis [37], fat infiltration [38], or muscle fiber branching [39]. While aponeurosis‐seeding based tracts could terminate mid‐muscle due to partial volume artifacts caused by fibrosis/fat replacement, voxel‐based seeding methods are more likely to uniformly sample the muscle regardless of fiber tract discontinuities, as fiber tracts will be initiated from different areas of the muscle.

### 4.5 Conclusions

We have implemented and evaluated several updated fiber‐tract seeding methods and tract quantification capabilities. Updated aponeurosis seeding procedures allow more accurate and robust tract propagation from seed points, as well as a broader range of structural quantification. The voxel‐based seeding methods implemented here produced quantification outcomes that differed slightly from the aponeurosis seeding method and are expected to accelerate high throughput analyses of multi‐muscle datasets. However, the differences in quantification outcomes indicate that the seeding method should be stated in skeletal muscle tractography studies. Also, we note that these conclusions are limited to the muscle architecture conditions evaluated here; therefore, the continued evaluation of these methods, in a wider range of muscle architectures, is warranted.

## Author Contributions

Conception and design of study: B.M.D. Data acquisition: B.M.D., R.P.G., C.A.L., and X.Z. Data analysis: B.M.D., R.P.G., C.A.L., and X.Z. Interpretation of data: B.M.D., R.P.G., C.A.L., and X.Z. Writing of manuscript: B.M.D. Editing and critical review of manuscript: R.P.G., C.A.L., and X.Z.

## Supporting information


**Figure S1:** Azimuthal muscle fiber orientations in the simulated muscle. White portions of the image show pixels contained within the muscle boundary mask. Interior black pixels show the central aponeurosis. Red lines show in‐plane muscle fiber orientations as lines connecting paired points along the aponeurosis and the outer muscle boundary.
**Figure S2:** Seed point rounding for aponeurosis seeding. Panel A illustrates the boundary between the muscle, illustrated as white‐shaded voxels, and its aponeurosis of muscle fiber insertion, illustrated as the gray voxels. The central voxel is labeled as {RN, CN} (Row N, Column N). The red line indicates points digitized to form the aponeurosis seeding mesh. The position of point P within voxel {RN, CN} is such that when the coordinates are rounded to integers for lookup from the imaging matrices, the point is shifted from the muscle region to the voxel {RN, CN + 1} in the aponeurosis region of the image. Because the mask value in the aponeurosis is set to zero, a tract would fail to initiate. Panel B illustrates the proposed procedure in which four sets of indices are created for lookup: {RN, CN}, {RN, CN + 1}, {RN + 1, CN}, and {RN + 1, CN + 1}. The solid lines indicate row and column combinations that would produce valid indices into the muscle region of the image mask. The coordinates producing the shortest distance are used to look up the initial tract propagation direction.
**Table S1:** MuscleDTI_Toolbox functions used in the present work. Files are located at https://github.com/bdamon/MuscleDTI_Toolbox.
**Figure S3:** Seed point distribution, VXL‐seeding condition. The seed points were placed at a frequency of 1 point/2 voxels in the row and column directions, within a once‐eroded muscle boundary mask. Dark blue portions of the images represent image regions outside of the muscle; yellow portions indicate seed points; and teal portions represent unseeded portions of the original muscle boundary mask.
**Figure S4:** Seed point distribution, Edge‐seeding condition. The seed points were placed one voxel interior to the outer edge, and one voxel exterior to the central aponeurosis, of a once‐eroded muscle boundary mask. Dark blue portions of the images represent image regions outside of the muscle; yellow portions indicate seed points; and teal portions represent unseeded portions of the original muscle boundary mask.

## Data Availability

The data that support the findings of this study are available from the corresponding author upon reasonable request. Per the IRB protocol, a Data Use Agreement must be established between the partnering institutions and the requesting investigator must document successful completion of human research ethics training.
