# Supplementary material for: A Comparison of Skeletal Muscle Diffusion Tensor Imaging Tractography Seeding Methods
Source: NMR Biomed. 2025 Oct 16;38(12):e70163. doi: 10.1002/nbm.70163 (PMC12529087; doi:10.1002/nbm.70163)
Supplement: Supplementary file 1 — Figure S1: Azimuthal muscle fiber orientations in the simulated muscle. White portions of the image show pixels contained within the muscle boundary mask. Interior black pixels show the central aponeurosis. Red lines show in‐plane muscle fiber orientations as lines connecting paired points along the aponeurosis and the outer muscle boundary. Figure S2: Seed point rounding for aponeurosis seeding. Panel A illustrates the boundary between the muscle, illustrated as white‐shaded voxels, and its aponeurosis of muscle fiber insertion, illustrated as the gray voxels. The central voxel is labeled as {RN, CN} (Row N, Column N). The red line indicates points digitized to form the aponeurosis seeding mesh. The position of point P within voxel {RN, CN} is such that when the coordinates are rounded to integers for lookup from the imaging matrices, the point is shifted from the muscle region to the voxel {RN, CN + 1} in the aponeurosis region of the image. Because the mask value in the aponeurosis is set to zero, a tract would fail to initiate. Panel B illustrates the proposed procedure in which four sets of indices are created for lookup: {RN, CN}, {RN, CN + 1}, {RN + 1, CN}, and {RN + 1, CN + 1}. The solid lines indicate row and column combinations that would produce valid indices into the muscle region of the image mask. The coordinates producing the shortest distance are used to look up the initial tract propagation direction. Table S1: MuscleDTI_Toolbox functions used in the present work. Files are located at https://github.com/bdamon/MuscleDTI_Toolbox. Figure S3: Seed point distribution, VXL‐seeding condition. The seed points were placed at a frequency of 1 point/2 voxels in the row and column directions, within a once‐eroded muscle boundary mask. Dark blue portions of the images represent image regions outside of the muscle; yellow portions indicate seed points; and teal portions represent unseeded portions of the original muscle boundary mask. Figure S4: Seed point distr [file NBM-38-e70163-s001.pdf]

S1. Detailed Methods: Definition of Model Fiber Orientations

The model tissue had an elliptical profile, with major and minor radii that varied as a function of slice number. To create this profile, a mask signal, S_M, was calculated as:

$$S_M = {r^2}/{a(s)} + {c^2}/{b(s)} \qquad \text{[S1]}$$

With *r* the distance from the image center in pixels along the row dimension and *c* the distance from the image center in pixels along the column dimension. *a* and *b* were empirically set in a slice number-dependent manner:

$$a = \begin{cases} 12s & s \leq 25 \\ 300 - 15 * |25 - s| & s > 25 \end{cases} \qquad \text{[S2a]}$$

$$b = \begin{cases} 6s & s \leq 25 \\ 150 - 10 * |25 - s| & s > 25 \end{cases} \qquad \text{[S2b]}$$

with *s* the slice number. A composite tissue mask was formed by assuming values $S_M > 1$ to lie outside of the tissue boundaries and values ≤ 1 to lie inside of the tissue boundaries. The aponeurosis was centrally located in the row and column directions, with a maximum size of three pixels in the column direction, a maximum size in the row direction that was eight pixels less than the corresponding muscle size for the given slice, and round anterior and posterior edges. The muscle boundary mask was defined as the subtraction of the aponeurosis boundary mask from the composite tissue mask.

To model the muscle fiber orientations, the in-plane (azimuthal) and through-plane (elevation) components were separately considered. The in-plane components were modeled as follows. Points defining the outer muscle boundary and the muscle aponeurosis boundary were identified using MATLAB's *bwboundaries( )* function. The minimum row/median column coordinates in each boundary were aligned. The vectors were resized to a length of 150 points and redundant points were eliminated. Corresponding points were connected using straight lines (Figure S1).

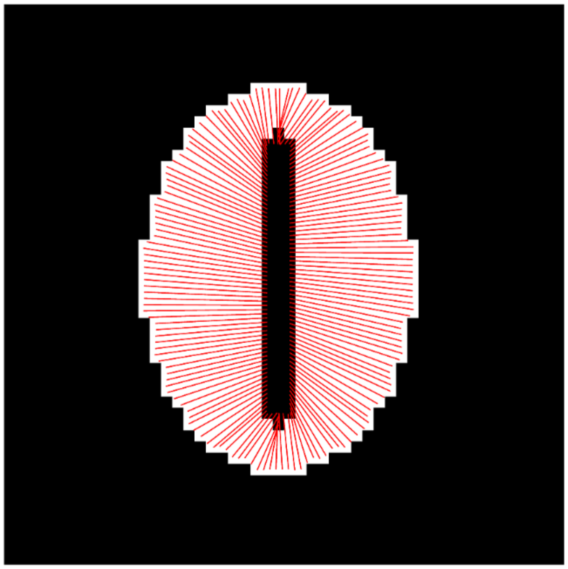

Figure S1. Azimuthal muscle fiber orientations in the simulated muscle. White portions of the image show pixels contained within the muscle boundary mask. Interior black pixels show the central aponeurosis. Red lines show in-plane muscle fiber orientations as lines connecting paired points along the aponeurosis and the outer muscle boundary.

The rise (Δ*y*) and run (Δ*x*) of each line was converted to a unit-length vector, [Δ*x* Δ*y*], that was used to describe the in-plane fiber orientation. Expressed in a spherical coordinate system, Δ*x* and Δ*y* describe the azimuthal angle, ϕ. To model the elevation angle θ, the Δ*z* component was defined as:

$$\Delta z = cot\left(\theta_B - \left(L_S + {}^{L_S}/_{6}\right)^2 - {}^{s}/_{10}\right) \quad \text{[S3]}$$

With θ_B the base elevation angle (=25°) and L_S the distance along the straight-line segment connecting the corresponding points. The second term on the right-hand side of Eq. S3 was set to cause θ to increase as a function of distance from the slice center, and the third term on the right-hand side of Eq. S3 was set to cause θ to increase as a function of slice number. This equation was solved at 20 linearly spaced points between the endpoints. At each intermediate point, the fiber orientation vector [Δ*x* Δ*y* Δ*z*] was constructed, converted to unit length, and taken as the local first eigenvector of the diffusion tensor, ε_1.

The high resolution of points along the muscle boundaries, and the solution of Eq. S3 at 20 points along the path between the corresponding points, caused there to be voxels with overlapping model muscle fibers. To form a net ε_1 at each voxel, the ε_{1x}, ε_{1y}, and ε_{1z} components of all muscle fibers passing through a voxel were separately summed, and the resulted summed vector was converted to unit length. Figure 1 in the main text illustrates the azimuthal and elevation angles corresponding to these net vectors.

S2. Updated Seed Point Rounding Procedures for Aponeurosis Seeding

Figure S2 illustrates the updated seed point rounding procedure.

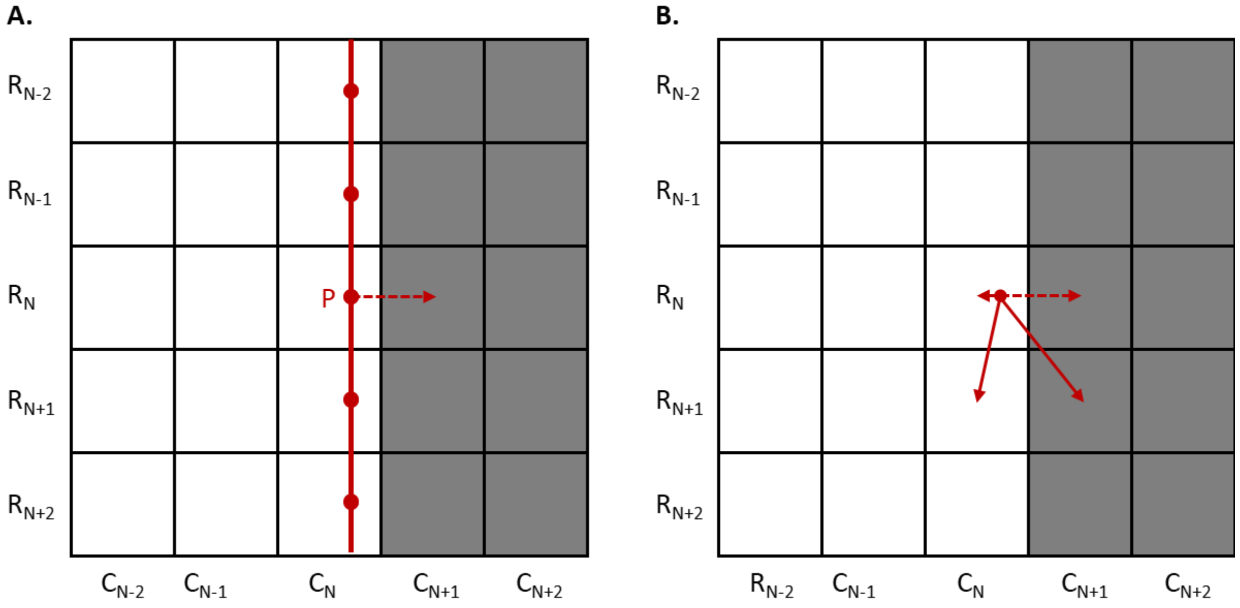

Figure S2. Seed point rounding for aponeurosis seeding. Panel A illustrates the boundary between the muscle, illustrated as white-shaded voxels, and its aponeurosis of muscle fiber insertion, illustrated as the gray voxels. The central voxel is labeled as $\{R_N, C_N\}$ (Row N, Column N). The red line indicates points digitized to form the aponeurosis seeding mesh. The position of point P within voxel $\{R_N, C_N\}$ is such that when the coordinates are rounded to integers for lookup from the imaging matrices, the point is shifted from the muscle region to the voxel $\{R_N, C_{N+1}\}$ in the aponeurosis region of the image. Because the mask value in the aponeurosis is set to zero, a tract would fail to initiate. Panel B illustrates the proposed procedure in which four sets of indices are created for lookup: $\{R_N, C_N\}$, $\{R_N, C_{N+1}\}$, $\{R_{N+1}, C_N\}$, and $\{R_{N+1}, C_{N+1}\}$. The solid lines indicate row and column combinations that would produce valid indices into the muscle region of the image mask. The coordinates producing the shortest distance are used to look up the initial tract propagation direction.

S3. MuscleDTI_Toolbox Modifications

Table S1 provides a list of function versions employed and their modifications from previous publications. The *far_stream_sampling( )* function is introduced in this publication.

Table S1. MuscleDTI_Toolbox functions used in the present work. Files are located at https://github.com/bdamon/MuscleDTI_Toolbox.

Function	Purpose	Version	Modifications from Prior Versions
define_roi( )	Uses aponeurosis mask to define aponeurosis seeding mesh	1.0.0	No modifications
fiber_track_v20( )	Propagates fiber tracts from seed points	2.0.0	Introduces several options for voxel-based seeding; for voxel seeding methods, eliminates points lying in the internal aponeurosis; removes fiber assignment by continuous tractography as a tracking algorithm option
fiber_smoother_v14( )	Performs polynomial smoothing for originally propagated fiber tracts	1.4.0	Minor bug fixes; adds ability to weight initial point in tract more heavily, improving fit quality; ensures compatibility with new fiber tract matrices following creation of new seeding capabilities
fiber_quantifier_v20( )	Obtains estimates of fiber tract orientations, length, and curvature	2.0.0	Adds calculation of α, β, and γ angles; ensures compatibility with new fiber tract matrices following creation of new seeding capabilities
fiber_visualizer_v12( )	Visualizes images with aponeurosis mesh, muscle boundary mask, and/or fiber tracts	1.2.0	Provides correct units for axis length scales
far_stream_sampling( )	Uniformly samples the muscle volume by creating a dataset with maximum inter-tract spacing	1.0	Newly introduced

S4. Seed Point Illustrations for Volume-based Voxel and Edge Seeding

Figure S3 illustrates the seed point distributions for the VXL condition.

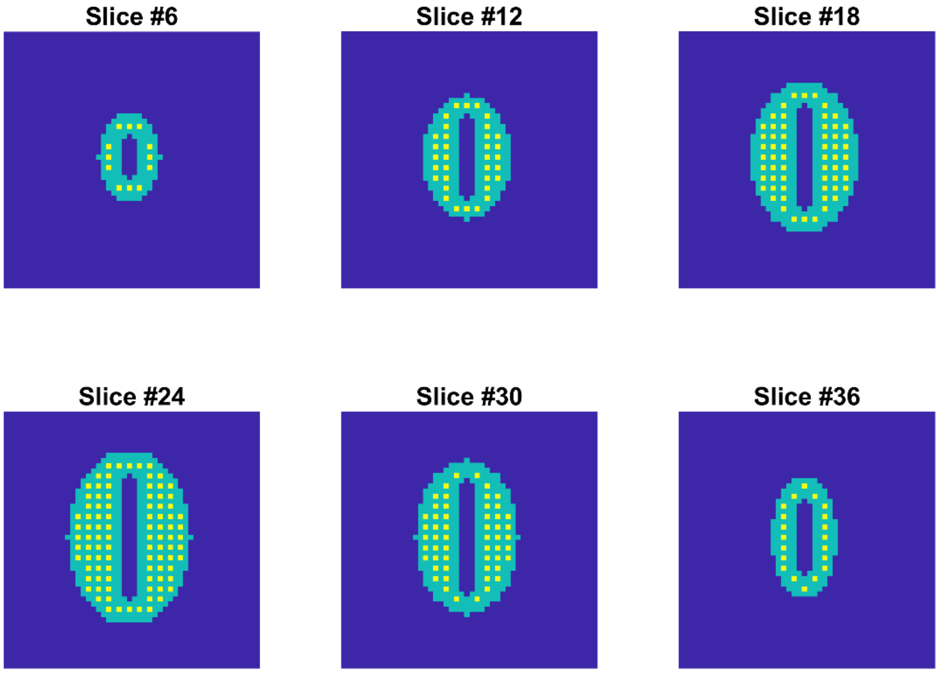

Figure S3. Seed point distribution, VXL-seeding condition. The seed points were placed at a frequency of 1 point/2 voxels in the row and column directions, within a once-eroded muscle boundary mask. Dark blue portions of the images represent image regions outside of the muscle; yellow portions indicate seed points; and teal portions represent unseeded portions of the original muscle boundary mask.

Figure S4 illustrates the seed point distributions for EDGE seeding.

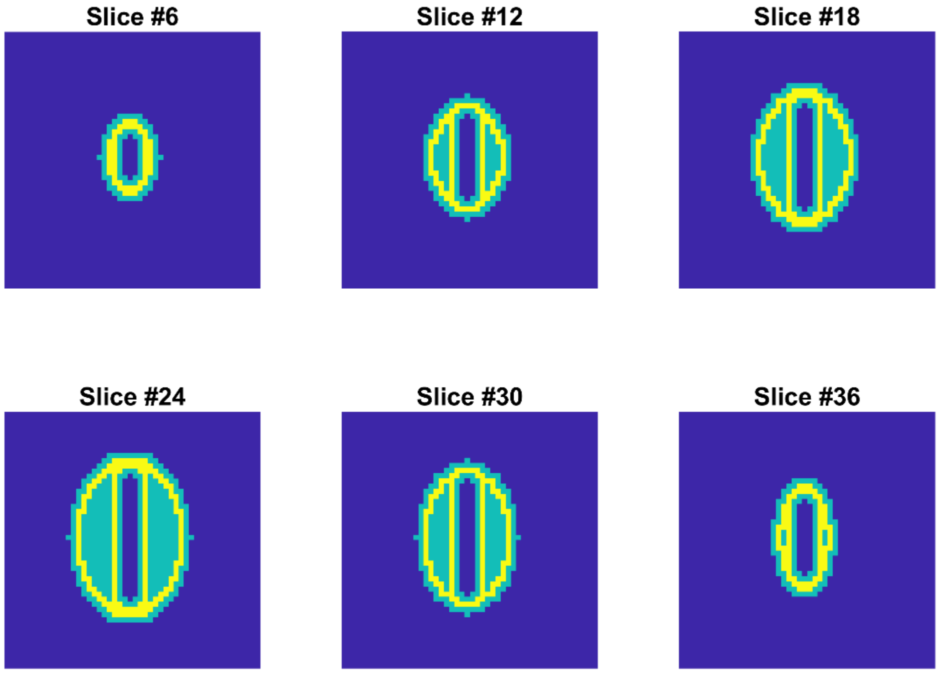

Figure S4. Seed point distribution, Edge-seeding condition. The seed points were placed one voxel interior to the outer edge, and one voxel exterior to the central aponeurosis, of a once-eroded muscle boundary mask. Dark blue portions of the images represent image regions outside of the muscle; yellow portions indicate seed points; and teal portions represent unseeded portions of the original muscle boundary mask.
